# Assessing the Optimum Level of Supplementation with Camelina Seeds in Ewes’ Diets to Improve Milk Quality

**DOI:** 10.3390/foods10092076

**Published:** 2021-09-02

**Authors:** Christos Christodoulou, Alexandros Mavrommatis, Christina Mitsiopoulou, George Symeon, Vasilis Dotas, Kyriaki Sotirakoglou, Basiliki Kotsampasi, Eleni Tsiplakou

**Affiliations:** 1 Laboratory of Nutritional Physiology and Feeding, Department of Animal Science, School of Animal Biosciences, Agricultural University of Athens, Iera Odos 75, 11855 Athens, Greece; c.christodoulou@aua.gr (C.C.); mavrommatis@aua.gr (A.M.); chr_mitsiopoulou@aua.gr (C.M.); 2 Research Institute of Animal Science, Hellenic Agricultural Organization—Demeter, 58100 Giannitsa, Greece; symeon@rias.gr (G.S.); vkotsampasi.arig@nagref.gr (B.K.); 3 Department of Animal Production, School of Agriculture, Aristotle University of Thessaloniki, 54124 Thessaloniki, Greece; vdotas@agro.auth.gr; 4 Laboratory of Mathematics and Statistics, Department of Natural Resources and Agricultural Engineering, School of Environment and Agricultural Engineering, Agricultural University of Athens, Iera Odos 75, 11855 Athens, Greece; sotirakoglou@aua.gr

**Keywords:** Camelina sativa, fatty acid profile, dairy, sheep, CLA, malondialdehyde, antioxidants, total antioxidant capacity

## Abstract

Camelina sativa seeds are rich in bioactive compounds such as polyunsaturated fatty acids (PUFA) and antioxidants, thus, their supplementation in ewes’ diets, may be an effective way to develop high nutritional dairy products. Therefore, the present study investigates the effect of the dietary inclusion of Camelina sativa seeds in ewes’ oxidative status and milk quality. Forty-eight dairy Chios ewes were divided into four homogenous groups and were fed individually. The concentrate of the control group (CON) had no inclusion of Camelina seeds, while the treatment groups (CSS6, CSS11, CSS16) were supplemented with 6%, 11%, and 16%, respectively. Including Camelina seeds in 6% and 11%, had no impact on milk performance, while in the CSS16, milk fat was significantly decreased compared to the CON. Supplementing Camelina seeds improved milk quality from a human health perspective by modifying the content of saturated fatty acid, the proportions of α-linolenic (C_18:3 n-3_), and C_18:2 cis-9, trans-11_ (CLA), and the ω6/ω3 ratio. Furthermore, the activity of catalase (CAT) was significantly increased in the CSS11 and CSS16, and superoxide dismutase (SOD) activity also significantly upsurged in the CSS16. Still, the levels of malondialdehyde (MDA) were significantly increased in the CSS11 compared to the CON and CSS6, and in the CSS16 compared to the CSS6. In CSS16, protein carbonyls were significantly increased. Finally, in the CSS-fed ewes, milk oxidative stability was fortified, as suggested by the modifications in the activities of SOD, CAT, and glutathione peroxidase (GSH-Px), in the antioxidant capacity, and the oxidative stress biomarkers. Consequently, the incorporation of 6% Camelina seeds in the concentrates of ewes improves milk’s fatty acid profile and oxidative status. However, more research is required regarding the possible negative effects of the constant consumption of Camelina seeds by ewes.

## 1. Introduction

In humans, the high intake of saturated fatty acids (SFA) has been associated with prospective risk of cardiovascular diseases, obesity, and metabolic syndrome [1], while that of monounsaturated (MUFA) and polyunsaturated fatty acids (PUFA) is associated with an improvement of dietary lipoprotein profiles, resulting in a plethora of benefits for human health [2]. Thus, manipulations in ruminants’ diets are studied aiming to alter milk fatty acids (FA) and manufacture functional dairy products with high economic and health potential. 

The inclusion of oilseeds rich in ω3 PUFA in ruminants’ diets appears to be a well-documented nutritional strategy to enrich ruminants’ milk with such bioactive molecules. Camelina is a low-input oilseed crop with superior nutrient efficiency that can grow with limited nitrogen fertilization [3]. Camelina seeds contain 40–44% crude protein and 39–47% fat depicting an interesting protein and energy source for high-yielding dairy animals. These aspects increase Camelina seeds and their by-products’ potential to partially substitute conventional feeds such as soya, thus preserving the biodiversity with simultaneous beneficial environmental outputs that are linked with vast soya cultivation on a global scale (e.g., deforestation, alternative use of land with fewer resources) [4]. However, due to antinutritional compounds that are presented in Camelina such as glucosinolates, tannins and erucic acid [5], the effect of constant consumption and its possible impact on the performance of animals should be also considered.

So far, the dietary supplementation with ω3 PUFA-rich Camelina Sativa seeds (CSS) resulted in an improvement on ewes’ milk FA profile from a human health point of view [6,7,8]. However, in these studies, the ewes were fed ad libitum on a group basis with pasture [6,8] or silage-based diets [7] which were also rich in ω3 PUFA (α-linolenic acid; C_18:3 n-3_ (ALA)). Furthermore, the narrow dietary inclusion levels of CSS (70 to 100 g/ewe/day) that were used in the aforementioned studies in combination with the type of forages (fresh; pastures, or silages) does not allow us to draw robust conclusions about the optimum supplementation level of ewes’ diets with CSS per se. Considering the above, more research is required to define the optimum supplementation level with CSS in ewes’ diets towards the enrichment of milk with PUFA. 

Even though the proportion of milk ω3 may be positively correlated with their dietary intake; extent levels of PUFA could adversely affect rumen fermentation, volatile fatty acids (VFA) production, and consequently animals’ performance. More specifically, the partial inhibition of principal rumen function, such as the biohydrogenation results in high accumulation of specific FAs such as C_18:2 trans-10, cis-12_, and C_18:1 trans-10_, which downregulate the lipogenesis in the mammary gland [9]. Thus, the dietary inclusion level of oilseeds rich in PUFA in ruminants should be holistically investigated, aiming to exclude the induction of adverse effects on animals’ performance, since plenty of cofactors such as animal species (goat, ewes, cows), lactation stage, oilseed, and forage type may synergistically act as well. 

Furthermore, supplementing high levels of oilseeds rich in PUFA may be a precursor of oxidative stress due to the high propensity of PUFA to oxidation. PUFA oxidation could trigger a cascade of prooxidant incidence disturbing the organism’s antioxidative balance and compromising milk oxidative status through the formation of detrimental aldehydes such as the malondialdehyde (MDA) [10]. Although high dietary PUFA levels could induce oxidative imbalances, prudent doses can activate antioxidant mechanisms in several cells [11]. In addition, Camelina oil is a pivotal source of tocopherols [12], a potent inhibitor of lipid peroxidation [13]. Considering the above, it is plausible to assume that the interaction of Camelina’s PUFA and antioxidants’ compounds are noteworthy for study under a more holistic lens. 

The objective of this work was to investigate the effects of dietary supplementation with CSS at three different levels (6%, 11%, and 16%) in milk yield and its chemical composition, and in FAs profile in ewes’ milk and blood plasma, having alfalfa hay and wheat straw as forages. At the same time, to evaluate the activity of key targeted antioxidant enzymes, such as glutathione peroxidase (GSH-Px), catalase (CAT), superoxide dismutase (SOD), glutathione reductase (GR), and glutathione transferase (GST) in ewes blood plasma, also GSH-Px, CAT, and SOD in ewes’ milk, the total antioxidant capacity [2,2′-Azino-bis (3-ethylbenzthiazoline-6-sulfonic acid) (ABTS) and the ferric reducing ability of both plasma and milk (FRAP)] and oxidative stress indicators such as malondialdehyde (MDA) and protein carbonyls (PCs) in both blood plasma and ewes’ milk.

## 2. Materials and Methods

### 2.1. Experimental Design and Diets

Forty-eight dairy Chios ewes, 2–4 years old and of comparable body weight (BW) (55.0 ± 6.5 kg) were kept at the Research Institute of Animal Science, Hellenic Agricultural Organization—Demeter (Giannitsa, Greece; 40°44′ N, 22°27′ E). Housing and care of the animals conformed to Ethical Committee guidelines of the Faculty of Animal Science (EU 63/2010; Council of the European Union 2010). Ewes were divided into four equal groups (*n* = 12) regarding BW, fat corrected (6%) milk yield (FCM) (1.85 ± 0.3 kg/d), days in milk (67 ± 8), and age (2–4 years old). Throughout the experimental period, ewes were kept in groups at a common stall and were fed individually.

The rations consisted of alfalfa hay, wheat straw, and concentrate and were formulated to be isonitrogenous with comparable caloric content amongst the groups. The daily feed intakes of the concentrate, alfalfa hay, and wheat straw were 1.5, 1.5, and 0.2 kg/ewe/day, respectively, and were offered at the same level, at each group, twice per day, after milking 07:00 and 17:00 (Table 1). The concentrate of the control group (CON) had no Camelina seeds, while in the three following groups (CSS6, CSS11, and CSS16), Camelina seeds were included at three different levels by partial substitution of soybean meal and maize grain (6, 11, and 16%, respectively) (Table 1). The concentrate consisted of maize grain, barley, wheat, sunflower meal, soybean meal, calcium carbonate, calcium phosphate, mineral and vitamin premix, and salt (Table 1). Before the official beginning of the experiment, there was a one-week adaptation period, mostly for the ewes to acclimatize to the new environment of individual feeding. Following the adaptation period, ewes were offered concentrate with the inclusion of three different levels of Camelina seeds. All animals had free access to fresh water. The main experimental period lasted 60 days.

### 2.2. Samples Collection 

Ewes were milked twice per day at 07:00 and 17:00 by a milking machine. Individual milk samples (*n* = 240) were collected at 0, 15, 30, 45, and 60 experimental days for milk chemical composition analysis and at 15, 30, 45, and 60 experimental days (*n* = 192) for milk FA profile determination and antioxidative status (five aliquots of 15 mL each). The stored milk aliquots were obtained after mixing a yield from the evening and the following morning milk on an identical percent volume (5%). Individual blood samples (*n* = 192) were also collected at 15, 30, 45, and 60 experimental days from the jugular vein of each ewe after the milking, prior to access on feeds. Approximately 10 mL of whole blood were immediately transferred to heparin-containing tubes (170 units heparin; BD Vacutainer, Plymouth, UK). Then, the blood samples were centrifuged (SL16R, Thermo Fisher Scientific, Waltham, MA, USA) at 2500 rpm for 15 min at 4 °C to separate plasma from the cells. Both the milk and blood samples were stored at −80 °C, before analysis. Furthermore, feed samples from alfalfa hay, wheat straw, and concentrate were collected at the beginning of the experiment, and every time a new concentrate was formulated. Samples of Camelina seeds were also collected for chemical analysis every time a new concentrate was formulated. 

### 2.3. Sample Analysis

#### 2.3.1. Feed Samples

Individual feed samples from alfalfa hay, wheat straw, Camelina seeds, and concentrates were collected and analyzed for organic matter (OM; Official Method 7.009), dry matter (DM; Official Method 7.007), and crude protein (CP; Official Method 7.016) according to AOAC [14]. Briefly, 0.5 g of 1 mm ground feedstuff was duplicate analyzed using a FOSS Kjeltec™ 8400 Analyzer Unit and a FOSS Digestion System DT220 (FOSS, Hillerød, Denmark) to estimate the nitrogen content, and then crude protein content was obtained by multiplying N × 6.25. Furthermore, ether extract was assayed based on Soxhlet [15] using 70 mL of petroleum ether (Sigma-Aldrich, St. Louis, MO, USA) per 1 g of 1 mm ground feedstuff for 50 min. Feed samples were also analyzed for neutral detergent fibre (NDF), assayed with a heat-stable amylase (Sigma-Aldrich, St. Louis, MO, USA) and acid detergent fibre (ADF), expressed exclusive of residual ash according to Van Soest et al. [16] using an ANKOM 2000 fiber analyzer and F57 filter bags (ANKOM Technology, Macedon, NY, USA) (Table 1). Feed samples were also analyzed for the determination of FA profile according to the method of O’ Fallon et al. [17] (Table 1).

#### 2.3.2. Milk Chemical Composition

Individual milk samples were analyzed for fat, protein, lactose, and solids-not-fat (SNF) using infrared spectroscopy (Milkoscan 6000; FOSS, Hillerød, Denmark), and for somatic cell counts (SCC) using Fossomatic 400 cell counter (FOSS, Hillerød, Denmark). Fat corrected- (FCM_6%_) and energy corrected- (ECM) milk yield was calculated using the following formulas:

Fat corrected milk (FCM) in 6% based on Equation (1).

FCM_6%_ = (0.28 + 0.12 × F) × M, (1)

where F = fat content (%) and M = milk yield in kg.

Energy corrected milk (ECM) yield based on Equation (2).

ECM = milk yield × (0.071 × fat (%) + 0.043 × protein (%) + 0.2224).(2)

#### 2.3.3. Fatty Acid Determination

The plasma fatty acid analysis was carried out according to the method of Bondia-Pons et al. [18]. The extraction and methylation of milk fatty acids were performed based on a two-step procedure as previously described by Tsiplakou et al. [19]. The FA profile was analyzed as described by Mavrommatis and Tsiplakou [20]. More specifically, an Agilent 6890 N gas chromatograph equipped with an HP-88 capillary column (60 m × 0.25 mm i.d. with 0.20 μm film thickness, Agilent) and a flame ionization detector, was used. A flame ionization detector (FID) temperature was set at 260 °C and the chromatographic analysis involved a temperature programmed run starting at 120 °C and held for 1 min. It was followed by two step-up ramps, one of 1.25 °C/min to 230 °C, and another of 10 °C/min to 240 °C and held for 3 min. Hydrogen was used as the carrier gas with a linear velocity set at 30 cm/s and helium as make up gas. Each peak was identified and quantified using a 37 component FAME mix standard (Supelco, Sigma Adrich Co., St. Louis, MO, USA). Additionally, extra standards were used for the C_18:2 cis-9, trans-11_, C_18:2 trans-10, cis-12_, and C_18:1 trans-11_ FA (Sigma Adrich Co., St. Louis, MO, USA). A Tricosanoic methyl ester (C_23:0_) was used as an internal standard for the chromatographic analysis (Fluka, Sigma Aldrich Co., St. Louis, MO, USA) for the milk samples and Tridecanoic (C_13:0_) for the blood plasma samples. The groups of FA were defined based on Tsiplakou et al. [21] as follow:

Short-Chain Saturated Fatty Acids (SCFA) = C_4:0_ + C_6:0_ + C_8:0_ + C_10:0_ + C_11:0_, (3)

Medium-Chain Saturated Fatty Acids (MCFA) = C_12:0_ + C_14:0_ + C_15:0_ + C_16:0_, (4)

Long-Chain Saturated Fatty Acids (LCFA) = C_17:0_ + C_18:0_ + C_20:0_ + C_22:0_, (5)

Mono-Unsaturated Fatty Acids (MUFA) = C_14:1_ + C_15:1_ + C_16:1 n-7_ + C_17:1 n-7_ + C_18:1 trans_ + C_18:1 trans-11_ + C_18:1 cis-9_ + C_20:1 n-9_, (6)

Poly-Unsaturated Fatty Acids (PUFA) = C_18:2 cis-9, trans-11_ + C_18:2n-6 cis_ + C_18:2 n-6 trans_ + C_18:3 n-3_ + C_18:3 n-6_ + C_20:3 n-3_ + C_20:4 n-6_, (7)

Saturated Fatty Acids (SFA) = SCFA + MCFA + LCFA,(8)

Unsaturated Fatty Acids (UFA) = PUFA + MUFA,(9)

Saturated/Unsaturated (S/U) = (SCFA + MCFA + LCFA)/(PUFA + MUFA),(10)

Atherogenic index (AI) = (C_12:0_ + 4 × C_14:0_ + C_16:0_)/(PUFA + MUFA), (11)

Thrombogenic index (TI) = (C_14:0_ + C_16:0_ + C_18:0_)/(0.5 × MUFA) + (0.5 × ω6 PUFA) + (3 × ω3 PUFA) + (ω3 PUFA/ω6 PUFA), (12)

Health promoting index (HPI) = (ω6 PUFA + ω3 PUFA + MUFA)/(C_12:0_ + 4 × C_14:0_ + C_16:0_). (13)

#### 2.3.4. Antioxidant Enzyme Activities and Oxidative Status Indicators

The assays for antioxidant enzyme activities, oxidative stress indicators, and the total antioxidant capacity were performed using a UV/Vis spectrophotometer (GENESYS 180, Thermo Fisher Scientific, Waltham, MA, USA) as previously described [22]. The GSTs activities were estimated by monitoring the conjunction of GSH to 1-chloro-2,4-dinitrobenzene (CDNT) at 340 nm. CAT activity was assessed using a commercial spectrophotometric kit (Catalase Assay Kit; CAT100, Sigma-Aldrich, St. Louis, MO, USA). GSH-Px activity was proportionally assayed by monitoring the decrease in the nicotinamide adenine dinucleotide phosphate (NADPH) absorbance at 340 nm, in the presence of H_2_O_2_. GR activity was determined by measuring the reduction of oxidized glutathione (GSSG) to reduce glutathione in presence of NADPH at 340 nm. SOD activity was recorded by monitoring the inhibition of cytochrome c oxidation at 550 nm. MDA was measured according to Nielsen et al. [23] with some modifications described by Tsiplakou et al. [22]. The protein carbonyls (PC) were assayed according to the method of Patsoukis et al. [24]. The ABTS [2,2′-azino-bis(3-ethylbenz-thiazoline-6-sulfonic acid] and the ferric reducing ability of plasma (FRAP) were used to assess the total antioxidant capacity by the decolorization of ABTS·+ cation radical at 734 nm and by monitoring the reduction of Fe^3+^ into Fe^2+^ using 2,4,6-tripyridyl-s-triazine at 593 nm respectively. The full assays and sample quantities are available in the Supplementary Materials. 

### 2.4. Statistical Analysis

The dataset was evaluated in SPSS.IBM software (v 24.0, IBM Corp., Armonk, NY, USA) and the results are depicted as mean ± standard error of means (SEM). The effect of dietary treatment between four groups was assessed by performing a GLM for repeated measures analysis of variance. The dietary treatments (D) (D = CON, CSS6, CSS11, and CSS16) were defined as the fixed factor and the sampling time (S) (0, 15, 30, 45, and 60 days for milk yield and chemical composition and 15, 30, 45, and 60 days for fatty acids and antioxidative status in blood plasma and milk) as the repeated measure, while their interactions (D × S) were also assessed, according to the following model:

Y_ijkl_ = µ + D_i_ + S_j_ + A_k_ + (D × S)_ij_ + e_ijkl_ (14)

where is $Y_{ijkl}$ the dependent variable, μ the overall mean, $D_{i}$ the effect of dietary treatment (*i* = 4; CON, CSS6, CSS11, and CSS16), $S_{j}$ the effect of sampling time (*j* = 5; 0, 15, 30, 45, and 60 days for milk yield and chemical composition or *j* = 4; 15, 30, 45, and 60 days for fatty acids and antioxidative status in blood plasma and milk), $A_{k}$ is the animal’s random effect, ${\left(D\times S\right)}_{ij}$ the interaction between dietary treatments and sampling time, and $e_{ijkl}$ the residual errors. A total of 240 observations (12 ewes × 4 dietary groups × 5 sampling times) emerged for milk yield and chemical composition while a total of 192 observations (12 ewes × 4 dietary groups × 4 sampling times) were obtained for fatty acids and antioxidative status in blood plasma and milk, respectively. Post-hoc analysis was applied with appropriate use of Tukey’s multiple range test. For all tests, the significance level was set at *p* = 0.05. Simplifying the visualization of these results, GraphPad Prism 6.0 (2012) depicted interleaved bars.

Discriminant analyses were also performed (variables were entered independent together; Figure 2A and using a stepwise method; Figure 2B) on milk fatty acids pooled data to establish those variables capable of distinguishing and classifying samples amongst the four dietary groups (CON, CSS6, CSS11, and CSS16). Wilk’s lambda (λ) criterion was used for assessing discriminant functions. Fifty-five variables for milk fatty acid profile were entered to create two models to distinguish the one hundred ninety-two samples of each case (4 groups × 12 ewes/group × 4 sampling time). Moreover, Pearson correlations were performed in the milk fatty acid profile featuring significant correlations between individual fatty acids.

## 3. Results

### 3.1. Feeds Fatty Acid Profile 

In the Camelina enriched concentrates, the proportions of, ALA, C_18:3 n-6_, C_20:0_, C_20:1 n-9_, C_20:2_, C_22:1 n-9_, and C_24:1 n-9_ were linearly increased in a dose-response. In addition, the proportions of C_14:0_, C_16:0_, C_16:1 n-7_, C_17:0_, C_17:1 n-7_, C_18:0_, C_18:1 cis-9_, and C_18:2 n-6 cis_ linearly decreased in a dose-dependent manner in the concentrates of the treated groups. Finally, the proportions of C_15:0_ and C_22:0_ were higher in the CSS concentrates, while the proportions of C_18:1 trans_, C_20:3 n-3_, and C_24:0_ were lower (Table 1). 

### 3.2. Milk Yield and Milk Chemical Composition

At the beginning of the trial (0th experimental day), there were no significant differences in milk yield and milk chemical composition among the four experimental groups. During the experimental period, and compared to the CON, milk yield showed a tendency to increase in the treated with Camelina seeds ewes, but it was not statistically significant (Table 2). Regarding milk chemical composition, milk fat was significantly decreased in the CSS16 compared to the CON and CSS11 (*p* = 0.046). Furthermore, total solids were found to be significantly elevated (*p* = 0.054) in the CSS11 compared to the CSS16. Fat yield, FCM, ECM, protein, protein yield, lactose, solids not fat (SNF), and somatic cell count (SCC) were not significantly affected (Table 2).

### 3.3. Blood Fatty Acids Profile

Significant differences in the FA profiles in blood plasma are depicted in Table 3. In Camelina-fed ewes, the proportions of several SFA were reduced. In detail, C_14:0_ was significantly reduced (*p* = 0.001) in the Camelina-fed ewes. In compliance, the proportion of C_15:0_ was reduced in the Camelina-fed ewes, but this reduction was significant (*p* = 0.025) only for the CSS16. Furthermore, the proportion of C_16:0_ was found to be significantly reduced (*p* = 0.018) in the CSS11, while the proportion of C_17:0_ was significantly reduced (*p* < 0.001) in the CSS6 and CSS11 compared to the CON and CSS16 groups. In addition, the proportions of C_16:1 n-7_ (*p* = 0.007), and C_18:1 cis-9_ (*p* = 0.003) were significantly reduced in the CSS16. The proportion of the C_18:2 n-6_ trans was significantly increased (*p* = 0.007) in the CSS16 compared to the CON and CSS11, while the proportion of C_18:2 n-6 cis_ was increased in the three CSS-fed ewes, but this increase was significant (*p* < 0.001) only in the CSS6 and CSS11. In addition, the proportion of C_20:3 n-3_ was significantly increased (*p* = 0.001) in the CSS6, the proportion of C_24:1 n-9_ was significantly enhanced (*p* < 0.001) in the CSS11 and CSS16 compared to the CSS6, while the proportion of C_22:6 n-3_ was significantly increased (*p* < 0.001) in the CSS11 and CSS16 compared to the CON and CSS6. Finally, the proportions of C_11:0_, C_17:1 n-7_, C_18:0_, C_18:1 trans_, C_18:1 trans-11_, ALA, C_20:3 n-6_, and C_22:2 n-6_ were not significantly affected. 

### 3.4. Milk Fatty Acids Profile

The inclusion of Camelina seed levels in ewes’ diets significantly (*p* < 0.001) reduced the SFA content (Table 4). In detail, the total SCFA and MCFA content and, more specifically the proportion of C_8:0_, C_10:0_, C_11:0_, C_12:0_, and C_16:0_ were linearly reduced (*p* < 0.001) in Camelina-fed ewes (Table 4). The MCFA content was significantly increased on the 30th sampling time compared to the 15th (*p* = 0.045). The proportions of C_6:0_ (*p* < 0.001) and C_15:0_ (*p* < 0.001) were significantly reduced in the CSS11 and CSS16, while that of C_14:0_ found significantly reduced (*p* = 0.005) in the CSS16. In contrast, the proportion of C_4:0_ was significantly increased (*p* = 0.174) in the CSS6, the proportion of C_17:0_ was significantly increased (*p* = 0.011) in the CSS6 compared to the CSS11, and the proportion of C_22:0_ was significantly increased (*p* < 0.001) in the CSS-fed ewes. Additionally, supplementing Camelina seeds in ewes’ diets significantly increased (*p* < 0.001) the proportion of C_18:0_ and C_20:0_ among the CSS groups and compared to the CON. Regarding C_18:0_, the same trend was reported on the 15th sampling time compared to the 30th and the 60th (*p* = 0.002).

In addition, the total content of MUFA was linearly increased (*p* < 0.001) among the Camelina-fed ewes (Table 4). In detail, supplementing ewes’ diets with Camelina seeds at 11%, significantly increased (*p* < 0.001) the proportion of C_18:1 trans-11_, while supplementing with 16% almost threefold significantly increased (*p* < 0.001) its proportion. However, milk C_18:1 trans-11_ proportion was highly accumulated at the 15th and the 60th compared to 45th experimental day (*p* = 0.046). In contrast, the proportions of C_14:1_ (*p* < 0.001), C_15:1_ (*p* = 0.001) and C_17:1 n-7_ (*p* < 0.001) were reduced in the CSS11 and CSS16, while the proportion of C_16:1 n-7_ was significantly reduced (*p* < 0.001) in the three CSS-fed ewes (Table 4). The proportions of total C_18:1 trans_ (*p* < 0.001), C_18:1 cis-9_ (*p* < 0.001), and C_20:1 n-9_ (*p* < 0.001) were linearly increased among the Camelina-fed ewes compared to the CON (Table 4). 

Furthermore, supplementing Camelina seeds in ewes’ diets linearly increased the total content of LCFA (*p* < 0.001), UFA (*p* < 0.001), and PUFA (*p* < 0.001), with the latter being even two-fold increased it in the CSS16 (Table 4). This increase in PUFA was reported through the four sampling times but was significant (*p* = 0.005) only on the final sampling, resulting in a significant interaction (*p* = 0.013) between the dietary treatment and sampling time. In details, the proportions of C_18:2 n-6 trans_ (*p* < 0.001), ALA (*p* < 0.001), C_18:2 cis-9, trans-11_ (CLA) (*p* < 0.001), C_18:2 trans-10, cis-12_ (CLA) (*p* < 0.001) and arachidonic acid (C_20:4 n-6_) (*p* < 0.001) were linearly increased among the Camelina-fed ewes and compared to the CON (Table 4). In fact, in the CSS16, the proportion of ALA was two-fold raised, while the proportion of C_18:2 cis-9, trans-11_ (CLA) was three-fold increased compared to the CON. For ALA, the same significant trend was observed on the 60th compared to the 30th experimental day (*p* = 0.010), while for C_18:2 cis-9, trans-11_ this increase was observed on the 45th and 60th compared to the 15th and the 30th experimental day (*p* < 0.001) and led to interaction between the dietary treatment and the sampling time (*p* = 0.003). Interestingly, the apparent transfer efficiency rate of ALA from the diets to milk was 5.8%, 4%, and 3,6% for the CSS6-, CSS11-, and CSS16-fed ewes, respectively. Still, the proportion of C_18:2 n-6 cis_ was significantly increased (*p* = 0.001) in the Camelina-fed ewes, while the proportion of C_20:3 n-3_ was significantly reduced (*p* < 0.001) in the CSS11 and CSS16 compared to the CON and CSS6 (Table 4). Finally, the SFA/UFA was linearly significantly decreased (*p* < 0.001) in the CSS-fed ewes compared to the CON.

Both ω6 and ω3 contents were linearly increased (*p* < 0.001) among the Camelina-fed ewes. Noteworthy, the ω6/ω3 was significantly reduced (*p* < 0.001) in the CSS-fed ewes (Table 4). In our study, atherogenicity (AI) and thrombogenic (TI) indexes were linearly decreased (*p* = 0.006 and *p* < 0.001, respectively) among the four dietary treatments (Table 4). In addition, the health-promoting index (HPI) was linearly improved among the four dietary treatments (*p* < 0.001). Concerning the Δ-9 desaturases indexes, C_14:1_/C_14:0_ was not affected. In contrast, C_16:1_/C_16:0_ was significantly increased (*p* = 0.024) in the CSS16, C_18:1 cis-9_/C_18:0_ was also increased (*p* < 0.001) in the CSS11 and CSS16, and C_18:2 cis-9, trans-11_/C_18:1 trans-11_ was linearly increased (*p* < 0.001) among the four dietary treatments as well (Table 4). 

**Figure 1:**
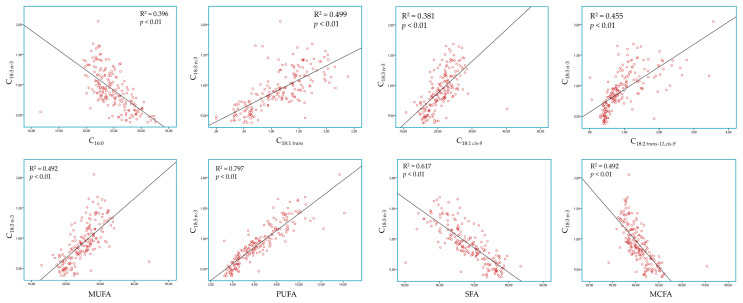
Pearson correlations concerning milk alpha linolenic acid (C18:3 n-3).

Pearson’s correlation led us to the conclusion that ALA was positively correlated with C_18:1 cis-9_ (R^2^ = 0.499; *p* < 0.01), C_18:1 trans_ (R^2^ = 0.381; *p* < 0.01), C_18:2 trans-11, cis-9_ (R^2^ = 0.455; *p* < 0.01), MUFA (R^2^ = 0.492; *p* < 0.01), and PUFA (R^2^ = 0.797; *p* < 0.01). On the other hand, ALA was negatively correlated with C_16:0_ (R^2^ = 0.396; *p* < 0.01), SFA (R^2^ = 0.617; *p* < 0.01), and MCFA (R^2^ = 0.492; *p* < 0.01) (Figure 1).

**Figure 2:**
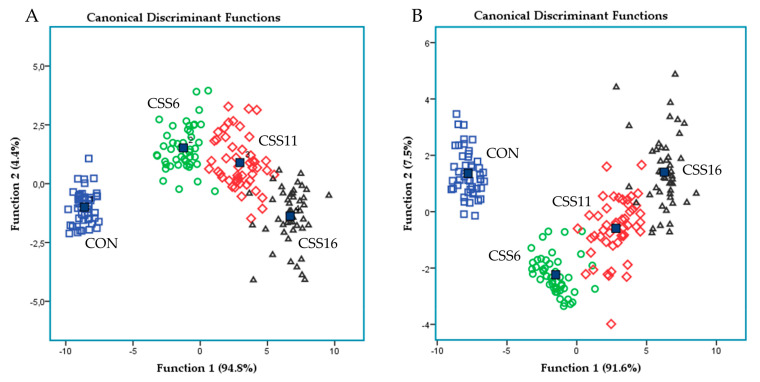
Discriminant plots separating (**A**) the four dietary treatments (CON; blue □, CSS6; green ○, CSS11; red ◇, and CSS16; black △) according to pooled data of four sampling times (15th, 30th, 45th, and 60th experimental day) that entered independent variables of milk individual fatty acids together, grouped fatty acids, fatty acids health indices, and Δ-9 desaturase indices and (**B**) discriminating the four dietary treatments (CON; blue □, CSS6; green ○, CSS11; red ◇, and CSS16; black △) based on a step-wise method.

Figure 2A,B depicts the discriminant plots of the four dietary treatments (CON; blue □, CSS6; green ○, CSS11; red ◇, and CSS16; black △) throughout the experimental period based on milk individual fatty acids, grouped fatty acids, fatty acids health indices, and Δ-9 desaturase indices. By inserting the independent variables together, the proportions of the samples that were correctly classified were 96.3%, while Wilks’s λ was observed at 0.009 for Function 1 (*p* < 0.001) and at 0.306 for Function 2 (*p* < 0.001) (Figure 2A). CON variables were significantly (Function 1) different from those of the Camelina-fed groups, while CSS groups were progressively allocated in the right half (Figure 2A). Applying a stepwise method aiming to avoid any multicollinearity, a higher correct classification was achieved (97.4%). Wilks’s λ was observed at 0.008 for Function 1 (*p* < 0.001) and at 0.238 for Function 2 (*p* < 0.001), while the proportion of C_20:0_, C_20:4 n-6_, C_18:1 trans_, C_22:0_, C_18:1 trans-11_, C_11:0_, C_18:2 n-6_, C_14:0_, C_18:0_, and C_18:2 trans-10, cis-12_ were the variables (10 out of 19) that contributed the most (Figure 2B). CON variables were significantly (Function 1) different from those of the Camelina-fed groups (Figure 2B). 

### 3.5. Blood Plasma and Milk Oxidative Status

The mean differences of the antioxidant enzyme activities, the total antioxidant capacity, and the oxidative status of blood plasma and milk are presented in Table 5. Concerning the antioxidant enzyme activities in ewe’s blood plasma, SOD activity was reported significantly higher (*p* = 0.014) in the CSS16. Still, an upward trend was observed in SOD activity from the 15th to the 60th experimental day (*p* < 0.001). Statistically significant enhanced activity (*p* = 0.006) was also observed in CAT in the CSS11- and CSS16-fed ewes, with the peak of activity being reported in the 30th sampling day (*p* = 0.052). However, we did not report significant differences in the activities of glutathione-related enzymes (GSH-Px, GR, and GST) (Table 5). Furthermore, the total antioxidant capacity based on the ABTS assay resulted in a significant increase (*p* < 0.001) in the CSS6 and CSS11 compared to the CON and CSS16. Additionally, ABTS depicted a significant peak on the 30th experimental day (*p* < 0.001), leading to a significant interaction (*p* < 0.001) between the dietary treatment and the sampling time. In contrast, in the CSS16, the total antioxidant capacity measured by the FRAP assay was significantly higher (*p* = 0.008) compared to the CON and CSS6, while the overall antioxidant capacity measured by FRAP was suppressed on the 45th experimental day (*p* = 0.001). Interestingly, regarding oxidative stress indicators, MDA demonstrated a significant increase (*p* < 0.001) in the CSS11 compared to the CSS6 and CON, while PCs were significantly increased (*p* < 0.001) in CSS16 compared to the rest groups. 

As for milk antioxidant enzyme activities, SOD activity was significantly increased (*p* = 0.019) in the CSS16. Similarly, SOD activity was significantly enhanced after the 15th experimental day (*p* < 0.001). Furthermore, CAT activity was significantly increased (*p* < 0.001) in the Camelina-fed ewes. More specifically, in the CSS16, CAT activity was significantly higher compared to CSS6 and CSS11, while even two-fold increase compared to the CON. Similarly, GSH-Px activity was also significantly higher (*p* < 0.001) in the Camelina-fed ewes. Interestingly, for both CAT and GSH-Px activity, the significant increase occurred in the 15th, 30th, and 60th compared to the 45th (*p* < 0.001) experimental day, leading to a significant interaction between the dietary treatment with the sampling time (*p* = 0.007, and *p* < 0.001 for CAT and GSH-Px, respectively). Concerning milk’s oxidative stress biomarkers, the highest MDA level was recorded in the CSS11, while the lowest was in the CSS16. Consequently, MDA was found to be significantly higher (*p* = 0.036) only for the CSS11 compared to the CSS16, with the same trend being reported in the 30th and 45th experimental days compared to the 15th (*p* = 0.023). Moreover, PCs were found to be significantly lower (*p* < 0.001) in the CSS16 compared to the rest groups. The PCs levels were significantly lower on the 15th experimental day compared to the rest (*p* < 0.001). Finally, we reported a statistically significant enhancement (*p* = 0.001) in the antioxidant capacity measured by ABTS assay in the CSS11 and CSS16. Moreover, FRAP values were significantly elevated in the Camelina-fed ewes and compared to the CON (*p* = 0.008). This increase in FRAP was linear amongst the four experimental groups and specifically in the CSS16 was significantly higher compared to the CSS6. For both ABTS and FRAP, the significant increase was reported in the first and the last sampling time compared to the 30th and 45th (*p* < 0.001, and *p* = 0.001 for ABTS and FRAP, respectively), which led to the significant interaction between the dietary treatments and the sampling time (*p* < 0.001) (Table 5).

## 4. Discussion

Without a doubt, supplementing Camelina seeds and their by-products in favor of soybean meal in animal diets can be considered a sustainable strategy in developing high-nutritional dairy products, simultaneously contributing against the environmental burden, and preserving biodiversity. However, caution should arise concerning the oxidative stability of both animal organisms and derived products. Since a diet supplemented with Camelina seeds probably exceeds the acceptable limits of dietary fat being offered to animals, and owing to a high PUFA content, the oxidative stability should also be determined to assess the optimum level of inclusion that would develop a high-quality product with desirable shelf-life longevity that respects the organism’s oxidative status. Furthermore, our study covered a 60-day experimental period, that ensured for each ewe the individual feeding had no adverse effect on their performance. Hence, we believe that our results could be a reference point for further assumptions and investigation to be conducted regarding the constant consumption of Camelina seeds that would partially substitute soybean meal in ewes’ diets and the possible impact of the antinutritional compounds that are presented.

### 4.1. The Inclusion of Camelina Seeds Had a Minor Impact on Milk Performance

Up until now, the inclusion of Camelina seeds in ruminant diets had a controversial impact on ruminants’ milk performance. In agreement with our findings, Hurtaud and Peyraud [25] and Mierlita et al. [6], did not report any significant difference concerning milk yield, when Camelina seeds and meal were included in both cows’ and ewes’ diets respectively. In contrast, Mierlita et al. [8] reported increased milk yield in ewes fed with pasture combined with a concentrate that included Camelina seeds (100 g/kg). Bayat et al. [26] reported that supplementing Camelina oil in dairy cows (60 g/kg DM), reduced milk yield and ECM yield. In concurrence with our study, Szumacher-Strabel et al. [27], reported significantly lower milk fat concentration after supplementing ewes’ diets with Camelina sativa cake. Contrary to our findings, offering Camelina seeds in ewes increased milk fat when animals were under grazing management (80 g/d) and in a study that was under a pasture-based dairy system (100 g/d) [6,8]. Bayat et al. [26] reported that the dietary supplementation with Camelina oil, reduced milk fat, milk protein, and lactose in dairy cows. In our study, the decreased milk fat content that was reported in the group with the highest level of supplementation, leads us to the conclusion that supplementing Camelina seeds up to 16%, may contribute to the so-called milk fat depression (MFD). Indeed, supplementing oilseeds in dairy cows tends to reduce milk fat content, causing MFD [9]. More specifically, C_18:2 trans-10, cis-12_ (CLA), may also act as an inhibitor of milk fat synthesis, and a high amount of milk total trans- FA concentration may also contribute to MFD [9]. However, more research is required referring to ewes and goats. What should also be noted is the fact that increased levels of PUFA lead to significant enhancement of ethanal synthesis during lactose–alcohol fermentation of sheep milk [28]. Furthermore, an increase in PUFA content could significantly affect the aroma profile of the kefir, by resulting in a loss of the animal odor in sheep milk kefir Cais-Sokolińska [28].

### 4.2. Supplementing Camelina Seeds Improves Ewes’ Milk Fatty Acid Profile

Camelina seed oil is richer in PUFA (73%) compared with other oilseeds like flaxseed, sunflower, soybean, and rapeseed [25]. The oil contains at average mainly ALA (~37%), C_18:2 n-6_ (15.2%), C_18:1 cis-9_ (~15%), C_20:1 n-9_ (15.5%), and C_22:1 n-9_ (~3%) [29]. Concerning milk FA profile, in compliance with our results, previous studies investigated the inclusion of Camelina seeds, oil, meal, cake, and expeller in ruminant diets and found decreased SFA content [6,7,8,25,26]. It has been reported that including oilseeds rich in C_18_ UFA in ruminant diets, reduces the proportion of SCFA and MCFA in milk [30]. Although oilseeds are responsible for dose-dependent decreases in the concentration of C_10:0_ to C_16:0_ FA [31], they also result in an increased proportion of the C_18:0_, C_18:1 cis-9_, and total C_18:1 trans_, which is also confirmed in the current study. In particular, the reduction of the SFA in favor of C_18:0_ can be justified by the fact that LCFAs are transferred mostly from blood, and they compete for the de novo lipogenesis of SCFA and MCFA in the mammary gland [32]. Still, the decreased proportions of C_14:0_, C_15:0_, C_16:0_, and C_17:0_ that were found in treated ewes’ milk samples, are also verified in their blood plasma.

Increases in C_18:1 cis-9_ and total C_18:1 trans_ were also observed through the supplementation of oilseeds in ruminant diets [31]. Indeed, supplementing ruminant diets with oilseeds rich in PUFA and especially in ALA may increase the C_18:1 trans_-FA, due to the lipolysis and biohydrogenation of C_18_ PUFA in the rumen [33]. Additionally, the reported increase in the proportion of C_18:1 trans-11_ that we observed, was also reported in ewes fed with Camelina seeds and meal [6,7,8,9]. In the same direction, in cows, Camelina seeds and meal, increased total MUFA content and the proportion of total C_18:1 trans_ [25,26]. Still, the reported increase in the proportion of C_18:1 trans-11_ in ovine milk occurs probably due to the action of the long-chain ω3 PUFA, which inhibits the final step of biohydrogenation [34]. 

Feeding whole oilseeds rather than the extracted oil is a more efficient way to enrich milk fat PUFA concentrations, due to the seed coat protecting the lipids from lipolysis and biohydrogenation in the rumen [33]. The ability to enrich ruminant milk with ALA was reported to be narrowed even when oilseeds rich in ALA are offered in ruminants [31]. However, in our study, ALA was linearly and significantly increased in treated with Camelina seeds ovine milk, indicating an efficient transfer in the three different studied levels of inclusion. Still, the apparent transfer efficiency of ALA was linearly decreased amongst the treated groups. This small increase in its proportion in milk is attributed to the biohydrogenation of PUFA in the rumen suggesting that most of the dietary ALA are exposed to extensive biohydrogenation in the rumen by the ruminal bacteria [35]. Noteworthy, although C_18:2 cis-9, trans-11_ (CLA) is the major isomer of CLA in ruminant milk and is an intermediate of the biohydrogenation of linoleic acid, its majority in milk fat is subjected to the desaturation of C_18:1 trans-11_ via the endogenous synthesis in the mammary gland, with the contribution of the Δ-9 desaturase enzyme. Therefore, the significant linear increase in the proportion of C_18:2 cis-9, trans-11_ (CLA) in ewes’ milk, is linked with the proportion of C_18:1 trans-11_. In compliance with our results, supplementing Camelina seeds increased the proportion of ALA and CLA in ovine milk [6,7,8,9]. In addition, in ewes that were under grazing management and were offered Camelina seeds (80 g/d) the proportions of ALA, and C_18:2 cis-9, trans-11_ (CLA) in milk, were significantly enhanced [6]. Similarly, ewes that were offered grass or maize silage with 70 g/kg DM Camelina seeds resulted in a significant increase in ALA and CLA proportions in milk [7]. Furthermore, combining pasture and concentrates that included Camelina seeds (100 g/kg in the concentrate), increased the proportion of ALA, C_18:2 cis-9, trans-11_ (CLA), DHA, and EPA in ewes’ milk [8]. In contrast, Hurtaud and Peyraud [25] reported that including Camelina in dairy cow diets did not significantly alter the proportion of ALA, while it increased that of CLA. Furthermore, supplementing 60 g of Camelina oil/kg of DM in dairy cows’ increased C18:2, ALA, CLA, total C_20:0_ and C_22:0_, PUFA, and total trans- FA proportions [26]. 

Noteworthy, ω3 PUFA, cannot be effectively synthesized by ruminant’s tissues. Their inclusion in human diets was linked with reducing the risk of heart attacks, coronary heart diseases, skin diseases, and arthritis. They can also reduce systemic inflammation and improve insulin resistance [36]. Besides, it is worth mentioning that C_18:2 cis-9, trans-11_ (CLA), which is also known as rumenic acid, gained major attention for its anticarcinogenic and antiatherogenic ability [37]. Therefore, the reported increased proportions of the aforementioned FAs in the treated with Camelina seed groups is considered a remarkable highlight towards developing a high-nutrition product from a consumer perspective.

In our study, a favorable outcome is also the fact that AI and ω6/ω3, were linearly decreased in the treatment groups and compared to the CON since higher AI and ω6/ω3 ratio are considered harmful for health [38]. Still, the reported significant alterations in the TI and the HPI were in favor of improving milk quality from a human health perspective.

In this study, the correlation amongst principal FA further confirms the aforementioned hypothesis regarding the competition of ALA and SFA. More specifically, the proportion of milk ALA was negatively correlated with C_16:0_, SFA, and MCFA. In contrast, the biohydrogenation of ALA to C_18:1 trans_ isomers was also confirmed by its positive correlation with C_18:1 trans_, C_18:1 cis-9_ and CLA. Notably, the correlation of ALA with C_18:1 cis-9_ and CLA does not indicate a direct relationship but unveils their formation through the stearic acid desaturation within the mammary gland.

### 4.3. Antioxidant Mechanisms Were Triggered to Preserve a Stable Oxidative Status in Ewes’ Blood Plasma While Milk Oxidative Status Was Promoted

The so-called oxidative stress occurs when there is an imbalance between the free radicals production and the antioxidant mechanisms. Therefore, since PUFA constitutes more than half of the total FA presented in Camelina, a common belief would suggest that this oil would demonstrate low oxidative stability [39]. Although high inclusion levels of ω3 long-chain PUFA in diets could induce oxidative imbalances as previously stated, prudent doses can activate antioxidant mechanisms in several cells [11] and thus restrain oxidative stress [40]. Camelina oil exhibits strong oxidative stability compared to flax oil, which is also rich in unsaturated fatty acids, but it is less stable compared with rapeseed, sesame, and sunflower oil [39]. As previously stated, Camelina oil is a remarkable source of γ-tocopherol [29]. Besides, it is a rich source of total phenolic compounds and flavonoids [41].

To the best of our knowledge, there is a lack of studies that investigate the optimum inclusion level of Camelina seeds that do not jeopardize ewes’ oxidative status and consequently their performance. In our study, the highest inclusion level (16%) increased the activity of SOD, which is the first line of defense against ROS [42]. This may be a precursor of a response to tolerate oxidation stress. More specifically, the increase in SOD activity may be a response towards neutralizing an increased concentration of superoxide anion (O_2_^●-^). Supplementing ω3 PUFA in human diets is in favor of formatting O_2_^●−^ [43], through the mitochondria respiratory. In agreement, palmitic acid in rat diets presents similar activity [44]. Besides, NADPH oxidase also provokes the concentration of O_2_^●−^ [45]. Increased SOD activity was also reported in goats’ blood plasma that was fed with high PUFA content [46]. Furthermore, supplementing whole sesame seeds in goats’ diets [47], also increased the SOD activity. The increased SOD activity in blood plasma and the increased levels of O_2_^●−^, catalyze efficiently the conversion of the latter to hydrogen peroxide (H_2_O_2_), a lesser unstable radical, and consequently results in an increased level of H_2_O_2_ and its by-products. 

Following the above, the H_2_O_2_ is detoxified by CAT and/or by GSH-Px. More specifically, CAT is responsible for the removal of the peroxides and their conversion into O_2_ [48]. In particular, the activity of CAT was found elevated in CSS11 and CSS16 compared to the CON, probably because of the increased substrate levels. However, the activity of CAT alone probably was unable to detoxify the H_2_O_2_ successfully, since GSH-Px activity did not differ significantly amongst the experimental groups. Increased CAT activity was also reported in goats fed with whole sesame seeds [47].

Owing to H_2_O_2_ action, higher production of the hydroxyl radical (OH^−^) may result, due to the Fenton reaction. This is supported in our case, through the higher reported concentration of MDA in the CSS11 and CSS16 blood. However, the lowest level of inclusion of Camelina seeds (6%), resulted in the lowest observed MDA level, compared to the CSS11 and CSS16. This may be related to low-grade oxidative stress that triggers the antioxidant mechanisms to neutralize lipid peroxidation in CSS6. Thus, the increased levels of PUFA in the two highest levels in ewes’ diets, may contribute to this upsurge in the MDA. Notably, MDA was determined as the most studied product of lipid peroxidation [23]. Nevertheless, the reported levels of MDA were within range compared to studies that were conducted with dairy ewes [49,50]. Likewise, PC was considered a biomarker of oxidative damage of proteins [51]. The reported level of PC in the experimental groups followed the same trend with those of MDA, which further supports the assumption of an oxidative response in the highest studied level of inclusion of Camelina seeds in ewes’ diets. As a matter of fact, a high dose of PUFA in goats’ diets, also resulted in increased MDA and PC levels [46]. Interestingly, the increased level of PC in the CSS16 group may be associated with the proteins’ oxidative damage by the oxygen-free radicals.

Contrary to the observations regarding the effect of the highest levels of inclusion of Camelina seeds in ewes’ oxidative status (blood), milk’s oxidative stability was strongly supported and not imperiled. Despite the significant linear increase that was previously reported in PUFA in the treated with Camelina seeds groups and compared to the CON, we achieved strong oxidative stability. In agreement with our results, Mierlita et al. [7] also reported that ewes being under a grass-silage-based diet and supplemented with Camelina seeds enhanced milk oxidative stability, which was measured with the Trolox equivalent antioxidant capacity. Nevertheless, we thoroughly approached milks’ oxidative stability mostly by determining not only the antioxidant capacity but also the activity of key targeted antioxidant enzymes that are involved in the antioxidant mechanisms, and oxidative stress biomarkers.

First and foremost, supplementing Camelina seeds in the three studied levels of inclusion boosted the key targeted antioxidant enzyme activities, and antioxidant capacity. Noteworthy, the oxidative stress biomarkers were not affected. This combined reported elevation in the activity of the studied antioxidant enzymes in ewes’ milk, alongside the significant increase in milk antioxidant capacity may result in a more stable product with better organoleptic characteristics and extensive shelf-life [21]. In a previous study, supplementing whole sesame seeds also boosted the antioxidant capacity that was measured with ABTS and FRAP assays in goats’ milk [47]. Moreover, supplementing both ewes and goats with PUFA also resulted in increased levels of CAT and FRAP [52]. In contrast, an infusion with high linolenic fatty acid in dairy cows, despite resulting in enhanced content of ω3 PUFA in milk, also negatively affected its oxidative stability [53], while supplementing ALA in vitro, was linked with increased free radical production in prostate cells compared with the supplementation with linoleic acid [54]. Finally, the fact that the oxidative stress biomarkers were not significantly altered in our study, further supports the assumption that milk’ oxidative stability was not jeopardized and there is a firm potential of developing a dairy product of high nutritional value to meet the consumer demands.

## 5. Conclusions

Camelina seeds demonstrate a noteworthy protein substitution for soybean meal in ewes’ diets. However, more research is needed to investigate its constant consumption, owing to the presence of antinutritive factors. Overall, supplementing ewes’ diets with the three inclusion levels (6, 11, and 16%), improved milk fatty acid profile from a human health point (increase PUFA and decrease SFA) as it was also reported in the literature. Nevertheless, the 6% inclusion level of Camelina seeds in the concentrates beneficially affected ewes’ oxidative status and milk oxidative stability. Therefore, enriching ewes’ diets with Camelina seeds (6%) portray an efficient protein substitution for soybean meal but also a decisive way to meet the global market trends, which are expressed by the increasing consumer demands of high nutritional and functional animal dairy products that promote human health.

## Figures and Tables

**Table 1:** Concentrates composition (g/kg), diet intake (g), daily nutrients intake (g/ewe), and feeds chemical composition and fatty acid profile (%).

Ingredients (%)	Concentrates								
	CON		CSS6		CSS11		CSS16		
Camelina seeds	0.0		6.0		11.0		16.0		
Maize grain	34.4		29.9		26.9		23.9		
Barley	20.0		20.0		20.0		20.0		
Wheat middlings	10.0		10.0		10.0		10.0		
Sunflower meal	16.0		18.0		18.0		18.0		
Soybean meal	15.5		12.0		10.0		8.0		
Premix mineral and vitamins	4.1		4.1		4.1		4.1		
Daily feed intake (g/ewe/d)	**Treatments**								
	**CON**		**CSS6**		**CSS11**		**CSS16**		
Wheat Straw	200		200		200		200		
Alfalfa Hay	1500		1500		1500		1500		
Concentrate	1500		1500		1500		1500		
Nutrients intake (g/day/ewe)	**Treatments**								
	**CON**		**CSS6**		**CSS11**		**CSS16**		
Dry Matter	2880		2874		2882		2888		
Crude Protein	601		600		602		620		
Ether Extract	40		80		107		133		
Neutral Detergent Fiber	1284		1285		1287		1301		
Acid Detergent Fiber	665		711		712		719		
	**Chemical composition (% DM)**								
	**Alfalfa hay**	**Wheat straw**		**CON con.**	**CSS6 con.**	**CSS11 con.**		**CSS16 con.**	
Dry Matter	89.40	92.80		90.24	89.85	90.38		90.74	
Crude Protein	20.00	0.48		20.03	19.96	20.09		21.30	
Ether Extract	0.28	0.16		2.36	5.00	6.80		8.55	
Neutral Detergent Fiber	60.60	72.80		15.29	15.34	15.52		16.42	
Acid Detergent Fiber	32.50	49.30		5.29	8.35	8.36		8.85	
	**Fatty acids composition (%)**								
Fatty acid	**Concentrates**				**Forages**				
	**CON con.**	**CSS6 con.**		**CSS11 con.**	**CSS16 con.**	**Alfalfa Hay**		**Wheat Straw**	
C_14:0_	0.18	0.16		0.15	0.10	2.35		6.16	
C_15:0_	0.00	0.04		0.04	0.03	0.68		0.83	
C_16:0_	15.71	10.18		8.63	7.68	43.53		33.38	
C_16:1 n-7_	0.22	0.14		0.12	0.11	2.99		0.00	
C_17:0_	0.21	0.10		0.09	0.08	0.83		0.00	
C_17:1 n-7_	0.41	0.10		0.08	0.04	0.00		0.00	
C_18:0_	2.94	2.46		2.28	2.17	6.79		4.28	
C_18:1 trans_	0.20	0.10		0.11	0.05	0.00		0.00	
C_18:1 cis-9_	20.65	17.89		17.84	17.35	3.01		9.00	
C_18:2 n-6 cis_	54.14	37.75		30.36	28.77	16.34		26.74	
C_18:3 n-6_	0.00	0.12		0.13	0.14	0.00		0.00	
C_20:0_	0.23	0.40		0.47	0.50	0.70		1.12	
C_18:3 n-3_	3.41	18.11		21.76	23.44	18.59		11.22	
C_20:1 n-9_	0.59	8.95		11.19	12.19	0.00		0.00	
C_20:2 n-6_	0.46	1.13		1.35	1.40	0.00		0.00	
C_22:0_	0.00	0.08		0.10	0.07	1.48		3.99	
C_20:3 n-3_	0.40	0.32		0.33	0.32	0.00		0.00	
C_22:1 n-9_	0.00	0.65		0.82	0.90	0.00		0.00	
C_24:0_	0.27	0.18		0.19	0.19	2.71		1.94	
C_24:1 n-9_	0.00	0.32		0.40	0.46	0.00		0.00	

CON: 0% Camelina seeds; CSS6: 6% Camelina seeds; CSS11: 11% Camelina seeds; CSS16: 16% Camelina seeds.

**Table 2:** Milk yield and chemical composition from ewes fed diets with different levels (CON, CSS6, CSS11, CSS16) with different levels of Camelina seeds (6%, 11%, and 16% of concentrate) throughout the experimental period (0, 15th, 30th, 45th, and 60th experimental days.

	Dietary Treatments (D)					Sampling Time (S)						Effect ^b^		
	Control	CSS6	CSS11	CSS16	SEM ^a^	0	15	30	45	60	SEM ^a^	D	S	D × S
Milk yield (g/d)	1705.0	1856.7	1873.7	1887.3	87.73	1973.1 ^C^	1869.8 ^B^	1866.7 ^BC^	1700.0 ^AB^	1743.8 ^BC^	53.05	0.433	<0.001	0.050
Fat (%)	5.89 ^b^	5.71 ^ab^	5.85 ^b^	5.35 ^a^	0.145	5.560 ^A^	6.021 ^B^	5.477 ^A^	5.976 ^B^	5.461 ^A^	0.103	0.046	<0.001	<0.001
Fat yield (g/d)	99.76	105.74	107.1	99.75	2.663	107.77 ^A^	112.12 ^A^	101.13 ^B^	100.35 ^B^	94.01 ^C^	2.888	0.511	<0.001	0.131
FCM _(6%)_ (g/d) ^c^	1678.1	1788.7	1809.2	1725.5	73.43	1845.7 ^BC^	1869.0 ^C^	1736.2 ^AB^	1684.6 ^A^	1616.4 ^A^	46.87	0.577	<0.001	0.252
ECM (g/d) ^d^	1470.84	1584.03	1633.53	1576.46	38.525	1648.12 ^A^	1658.51 ^A^	1562.62 ^B^	1496.42 ^C^	1465.40 ^C^	42.968	0.424	<0.001	0.193
Protein (%)	5.218	5.285	5.435	5.236	0.072	5.291 ^AB^	5.377 ^B^	5.233 ^A^	5.267 ^AB^	5.299 ^AB^	0.044	0.145	0.010	0.008
Protein yield (g/d)	89.15	97.76	101.26	98.60	2.487	103.93 ^A^	100.38 ^AB^	97.56 ^B^	89.41 ^C^	92.17 ^C^	2.796	0.267	<0.001	0.067
Lactose (%)	4.943	5.002	5.044	5.028	0.047	5.075 ^C^	5.003 ^AB^	4.971 ^AB^	4.949 ^A^	5.022 ^BC^	0.027	0.456	<0.001	0.011
SCC (1000/mL) ^e^	349.72	601.62	454.68	352.87	168.56	204.50 ^a^	599.63 ^B^	496.40 ^AB^	415.84 ^AB^	482.25 ^AB^	101.55	0.689	0.011	0.502
Total Solids (%)	16.73 ^ab^	16.56 ^ab^	16.90 ^b^	16.18 ^a^	0.185	16.49	16.97	16.25	16.76	16.48	0.120	0.054	<0.001	<0.001
Solis Not Fat (%)	10.84	10.86	11.05	10.83	0.078	10.93b ^c^	10.95 ^C^	10.78 ^A^	10.79 ^AB^	11.02 ^C^	0.052	0.165	<0.001	<0.001

Means with different superscript letters (a, b, c) between dietary groups and (A, B, C, D) between sampling time points differ significantly. ^a^ SEM: Standard error of the means. ^b^ Effect: The dietary treatment (D), time (S), and the interaction between dietary treatment × time (D × S) effects were analyzed by ANOVA using a general linear model (GLM) for repeated measures, and post-hoc analysis was performed with appropriate use of Tukey’s multiple range test. ^c^ Fat corrected milk (FCM) in 6%, ^d^ Energy corrected milk (ECM) yield, ^e^ Somatic Cell Counts.

**Table 3:** The mean individual fatty acids (FA) (% of total FA) in the blood plasma of ewes fed diets (CON, CSS6, CSS11, and CSS16) with different levels of Camelina seeds (6%, 11%, and 16% of concentrate) throughout the experimental period (15th, 30th, 45th and 60th experimental days).

Fatty Acid	Dietary Treatments (D)					Sampling Time (S)					Effect ^b^		
	CON	CSS6	CSS11	CSS16	SEM ^a^	15	30	45	60	SEM ^a^	D	S	D × S
C_11:0_	0.29	0.24	0.59	0.31	0.29	0.53 ^C^	0.21 ^A^	0.33 ^B^	0.36 ^B^	0.55	<0.001	0.001	<0.001
C_14:0_	1.02 ^b^	0.70 ^a^	0.65 ^a^	0.64 ^a^	0.34	0.90 ^B^	0.69 ^A^	0.77 ^AB^	0.64 ^A^	0.61	0.001	0.022	0.174
C_15:0_	0.37 ^b^	0.29 ^ab^	0.32 ^ab^	0.17 ^a^	0.19	0.29	0.26	0.25	0.35	0.37	0.025	0.273	0.393
C_16:0_	21.46 ^b^	20.57 ^ab^	19.83 ^a^	20.92 ^ab^	0.19	22.63 ^C^	20.53 ^B^	20.34 ^AB^	19.28 ^A^	0.34	0.018	<0.001	0.001
C_16:1 n-7_	1.06 ^b^	0.76 ^ab^	0.79 ^ab^	0.55 ^a^	0.42	1.12 ^B^	0.66 ^A^	0.67 ^A^	0.70 ^A^	0.79	0.007	0.001	0.655
C_17:0_	2.40 ^b^	1.73 ^a^	1.39 ^a^	2.66 ^b^	0.10	2.41	2.21	2.15	1.42	0.25	<0.001	0.006	<0.001
C_17:1 n-7_	0.02	0.05	0.02	0.05	0.01	0.03	0.04	0.06	0.02	0.01	0.351	0.518	0.275
C_18:0_	21.10	21.10	20.44	20.70	0.34	20.62 ^AB^	20.03 ^A^	20.65 ^AB^	22.05 ^B^	0.60	0.849	0.044	0.485
C_18:1 trans_	0.15	0.19	0.29	0.27	0.03	0.04 ^A^	0.17 ^BC^	0.27 ^C^	0.42 ^D^	0.39	0.056	<0.001	0.047
C_18:1 trans-11_	1.01	0.88	0.89	0.83	0.04	0.57 ^A^	0.81 ^B^	0.98 ^B^	1.25 ^C^	0.07	0.583	<0.001	0.024
C_18:1 cis-9_	15.91 ^b^	15.03 ^ab^	14.66 ^ab^	12.55 ^a^	0.30	14.40	13.75	14.84	15.17	0.59	0.003	0.356	0.020
C_18:2 n-6 trans_	0.03 ^a^	0.05 ^ab^	0.02 ^a^	0.10 ^b^	0.05	0.00 ^A^	0.00 ^A^	0.04 ^B^	0.17 ^C^	0.13	0.007	<0.001	0.014
C_18:2 n-6 cis_	19.26 ^a^	23.13 ^b^	21.66 ^b^	20.98 ^ab^	0.27	22.51 ^C^	19.97 ^A^	20.99 ^AB^	21.57 ^BC^	0.54	<0.001	0.002	0.028
C_18:3 n-3_	2.71	3.28	3.04	3.00	0.09	1.97 ^A^	2.67 ^B^	3.31 ^C^	4.07 ^D^	0.31	0.098	<0.001	0.062
C_20:3 n-6_	0.18	0.06	0.13	0.17	0.02	0.07 ^A^	0.07 ^A^	0.16 ^B^	0.24 ^B^	0.15	0.428	0.044	0.101
C_20:3 n-3_	3.21 ^a^	3.80 ^b^	3.36 ^a^	3.20 ^a^	0.08	4.10 ^D^	3.67 ^C^	3.10 ^B^	2.70 ^A^	0.14	0.001	<0.001	0.037
C_22:2 n-6_	0.84	0.94	0.92	0.84	0.03	0.58 ^A^	0.88 ^B^	0.98 ^BC^	1.11 ^C^	0.89	0.486	<0.001	0.728
C_24:1 n-9_	8.24 ^ab^	6.44 ^a^	9.85 ^b^	10.80 ^bc^	0.34	6.98 ^A^	12.20 ^C^	8.82 ^B^	7.34 ^A^	0.62	<0.001	<0.001	<0.001
C_22:6 n-3_	0.47 ^a^	0.61 ^a^	1.00 ^b^	1.02 ^b^	0.05	0.16 ^A^	1.04 ^B^	0.98 ^B^	0.92 ^B^	0.07	<0.001	<0.001	<0.001

Means with different superscript letters (a, b, c) between dietary groups and (A, B, C, D) between sampling time points differ significantly. ^a^ SEM: Standard error of the means. ^b^ Effect: The dietary treatment (D), time (S), and the interaction between dietary treatment × time (D × S) effects were analyzed by ANOVA using a general linear model (GLM) for repeated measures, and post-hoc analysis was performed with appropriate use of Tukey’s multiple range test.

**Table 4:** The mean individual fatty acids (FA) (% of total FA), grouped FA, FA health indices, and Δ-9 desaturase indices in the milk of ewes fed diets (CON, CSS6, CSS11, and CSS16) with different levels of Camelina seeds (6%, 11%, and 16% of concentrate) throughout the experimental period (15th, 30th, 45th and 60th experimental days).

Fatty acid	Dietary Treatment (D)					Sampling Time (S)					Effect^b^		
	CON	CSS6	CSS11	CSS16	SEM ^a^	15	30	45	60	SEM ^a^	D	S	D × S
C_4:0_	4.23 ^a^	4.45 ^b^	4.32 ^ab^	4.40 ^ab^	0.070	4.48 ^B^	4.20 ^A^	4.40 ^AB^	4.32 ^AB^	0.069	0.174	0.042	<0.001
C_6:0_	3.33 ^c^	3.18 ^c^	2.84 ^b^	2.29 ^a^	0.053	2.96	2.84	2.93	2.91	0.052	<0.001	0.476	<0.001
C_8:0_	3.08 ^d^	2.80 ^c^	2.39 ^b^	1.72 ^a^	0.057	2.54	2.48	2.49	2.48	0.058	<0.001	0.872	0.079
C_10:0_	9.53 ^d^	7.88 ^c^	6.56 ^b^	4.55 ^a^	0.187	7.17	7.13	7.08	7.15	0.176	<0.001	0.981	0.077
C_11:0_	0.37 ^d^	0.32 ^c^	0.28 ^b^	0.19 ^a^	0.009	0.27 ^A^	0.29 ^AB^	0.29 ^AB^	0.31 ^B^	0.009	<0.001	0.010	0.093
C_12:0_	5.15 ^d^	4.22 ^c^	3.72 ^b^	2.91 ^a^	0.099	3.97	4.09	3.80	3.94	0.089	<0.001	0.334	0.109
C_14:0_	12.64 ^b^	11.43 ^ab^	11.92 ^b^	10.02 ^a^	0.504	11.00	12.49	11.04	11.48	0.380	0.005	0.161	0.211
C_14:1_	0.42 ^c^	0.40 ^bc^	0.38 ^ab^	0.37 ^a^	0.007	0.37 ^A^	0.40 ^B^	0.41 ^B^	0.38 ^AB^	0.008	<0.001	0.002	0.174
C_15:0_	0.92 ^b^	0.89 ^b^	0.81 ^a^	0.80 ^a^	0.014	0.83 ^A^	0.89 ^B^	0.86 ^AB^	0.83 ^A^	0.014	<0.001	0.006	0.889
C_15:1_	0.28 ^b^	0.28 ^b^	0.25 ^a^	0.25 ^a^	0.007	0.27 ^B^	0.28 ^B^	0.28 ^B^	0.24 ^A^	0.007	0.001	<0.001	0.399
C_16:0_	28.55 ^d^	24.95 ^c^	23.40 ^b^	22.26 ^a^	0.271	24.27 ^A^	24.39 ^A^	25.50 ^B^	24.99 ^AB^	0.280	<0.001	0.011	0.500
C_16:1 n-7_	1.07 ^b^	0.96 ^a^	0.90 ^a^	0.92 ^a^	0.028	0.87 ^A^	0.91 ^AB^	0.99 ^BC^	1.07 ^C^	0.028	<0.001	<0.001	0.004
C_17:0_	0.52 ^ab^	0.53 ^b^	0.49 ^a^	0.50 ^ab^	0.009	0.54 ^C^	0.52 ^BC^	0.49 ^AB^	0.48 ^A^	0.010	0.011	<0.001	0.019
C_17:1 n-7_	0.25 ^b^	0.25 ^b^	0.21 ^a^	0.20 ^a^	0.007	0.23	0.23	0.23	0.22	0.007	<0.001	0.525	0.014
C_18:0_	7.68 ^a^	8.86 ^b^	9.10 ^b^	9.96 ^c^	0.119	9.42 ^B^	8.83 ^A^	9.09 ^AB^	8.26 ^A^	0.233	<0.001	0.002	0.602
C_18:1 trans_	0.52 ^a^	1.07 ^b^	1.41 ^b^	1.57 ^b^	0.046	1.12 ^AB^	1.20 ^B^	1.15 ^AB^	1.08 ^A^	0.039	<0.001	0.169	0.021
C_18:1 trans-11_	0.69 ^a^	0.86 ^ab^	1.13 ^b^	1.85 ^c^	0.105	1.27 ^B^	1.01 ^AB^	0.95 ^A^	1.31 ^B^	0.084	<0.001	0.046	0.015
C_18:1 cis-9_	16.58 ^a^	20.38 ^b^	21.77 ^c^	24.86 ^d^	0.279	21.34	20.59	20.80	20.85	0.553	<0.001	0.509	0.219
C_18:2 n-6 trans_	0.19 ^a^	0.55 ^b^	0.87 ^c^	1.21 ^d^	0.030	0.68 ^A^	0.70 ^AB^	0.65 ^A^	0.80 ^B^	0.030	<0.001	0.011	0.082
C_18:2 n-6 cis_	2.70 ^a^	2.90 ^b^	2.95 ^b^	3.09 ^b^	0.064	2.90	2.88	2.91	2.95	0.058	0.001	0.849	0.218
C_20:0_	0.11 ^a^	0.58 ^b^	0.85 ^c^	1.12 ^d^	0.017	0.65	0.67	0.66	0.68	0.018	<0.001	0.605	0.006
C_18:3 n-3_	0.54 ^a^	0.84 ^b^	1.04 ^c^	1.26 ^d^	0.028	0.92 ^AB^	0.87 ^A^	0.90 ^AB^	0.97 ^B^	0.028	<0.001	0.010	0.361
C_18:2 cis-9, trans-11_	0.45 ^a^	0.68 ^b^	1.03 ^c^	1.65 ^d^	0.048	0.82 ^A^	0.85 ^A^	1.06 ^B^	1.08 ^B^	0.05	<0.001	<0.001	0.003
C_20:1 n-9_	0.00 ^a^	0.37 ^b^	0.63 ^c^	0.88 ^d^	0.036	0.57 ^C^	0.59 ^C^	0.28 ^A^	0.44 ^B^	0.032	<0.001	<0.001	<0.001
C_18:2 trans-10, cis-12_	0.00 ^a^	0.00 ^a^	0.01 ^b^	0.06 ^b^	0.006	0.004 ^A^	0.03 ^B^	0.005 ^A^	0.03 ^B^	0.005	<0.001	<0.001	<0.001
C_22:0_	0.09 ^a^	0.20 ^b^	0.19 ^b^	0.25 ^c^	0.010	0.14 ^A^	0.20 ^BC^	0.21 ^C^	0.17 ^B^	0.009	<0.001	<0.001	<0.001
C_20:3 n-__3_	0.23 ^b^	0.21 ^b^	0.20 ^a^	0.18 ^a^	0.004	0.22 ^B^	0.20 ^A^	0.20 ^A^	0.20 ^A^	0.004	<0.001	0.001	0.442
C_20:4__n-6_	0.00 ^a^	0.02 ^a^	0.31 ^b^	0.61 ^c^	0.015	0.18 ^A^	0.22 ^AB^	0.25 ^BC^	0.29 ^C^	0.016	<0.001	<0.001	<0.001
Grouped Fatty Acids													
SCFA	20.54 ^d^	18.62 ^c^	16.39 ^b^	13.15 ^a^	0.303	17.40	16.94	17.18	17.18	0.300	<0.001	0.744	0.001
MCFA	47.28 ^d^	41.51^c^	39.85 ^b^	36.00 ^a^	0.420	40.09 ^A^	41.86 ^B^	41.32 ^AB^	41.36 ^AB^	0.442	<0.001	0.075	0.045
LCFA	8.19 ^a^	9.80 ^b^	10.45 ^c^	11.60 ^d^	0.207	10.61 ^B^	9.88 ^AB^	10.12 ^AB^	9.43 ^A^	0.218	<0.001	0.004	0.488
MUFA	19.78 ^a^	24.68 ^b^	26.68 ^c^	30.90 ^d^	0.349	26.02	25.36	25.18	25.49	0.694	<0.001	0.464	0.003
PUFA	4.12 ^a^	5.19 ^b^	6.44 ^c^	8.11 ^d^	0.129	5.74 ^A^	5.76 ^A^	5.98 ^A^	6.38 ^B^	0.254	<0.001	0.005	0.013
SFA	76.01 ^d^	69.93 ^c^	66.69 ^b^	60.75 ^a^	0.412	68.10	68.68	68.62	67.97	0.440	<0.001	0.598	0.001
UFA	23.90 ^a^	29.87 ^b^	33.12 ^c^	39.01 ^d^	0.412	31.76	31.12	31.16	31.86	0.438	<0.001	0.526	0.001
SFA/UFA	3.18 ^d^	2.34 ^c^	2.01 ^b^	1.56 ^a^	0.048	2.25	2.37	2.29	2.31	0.049	<0.001	0.504	0.005
ω6	2.91 ^a^	3.46 ^b^	4.14 ^c^	4.91 ^d^	0.069	3.76 ^A^	3.79 ^A^	3.82 ^A^	4.04 ^B^	0.136	<0.001	0.063	0.005
ω3	0.77 ^a^	1.05 ^b^	1.24 ^c^	1.44 ^d^	0.023	1.14 ^AB^	1.07 ^A^	1.10 ^A^	1.19 ^B^	0.361	<0.001	0.024	0.341
ω6/ω3	3.78 ^b^	3.30 ^a^	3.34 ^a^	3.41 ^a^	0.038	3.39 ^A^	3.59 ^B^	3.56 ^AB^	3.46 ^AB^	0.074	<0.001	0.189	0.628
Fatty Acids Health Indices													
AI	3.55 ^a^	2.54 ^b^	2.16 ^c^	1.70 ^d^	0.056	2.40 ^A^	2.53 ^B^	2.46 ^AB^	2.56 ^B^	0.555	0.006	0.101	0.003
TI	3.52 ^c^	2.60 ^b^	2.36 ^b^	1.90 ^a^	0.054	2.51	2.69	2.61	2.56	0.107	<0.001	0.294	0.040
HPI	0.28 ^a^	0.39 ^b^	0.46 ^c^	0.58 ^d^	0.010	0.44 ^A^	0.41 ^T^	0.42 ^A^	0.43 ^A^	0.020	<0.001	0.493	0.005
∆−9 Desaturase Indices													
C_14:1_/C_14:0_	0.033	0.035	0.032	0.037	0.002	0.03	0.04	0.03	0.04	0.002	0.520	0.198	0.526
C_16:1_/C_16:0_	0.037 ^a^	0.038 ^ab^	0.038 ^ab^	0.041 ^b^	0.001	0.036 ^A^	0.037 ^A^	0.038 ^AB^	0.042 ^B^	0.001	0.024	<0.001	0.010
C_18:1 cis-9_/C_18:0_	2.16 ^a^	2.30 ^ab^	2.39 ^bc^	2.50 ^c^	0.263	2.28 ^A^	2.35 ^A^	2.34 ^A^	2.59 ^B^	0.177	<0.001	0.366	0.388
C_18:2 cis-9 trans-11_/C_18:1 trans-11_	0.65 ^a^	0.79 ^b^	0.91 ^d^	0,89 ^c^	0.025	0.64 ^A^	0.83 ^B^	1.06 ^C^	0.94 ^B^	0.029	<0.001	<0.001	<0.001

Means with different superscript letters (a, b, c) between dietary groups and (A, B, C, D) between sampling time points differ significantly. ^a^ SEM: Standard error of the means. ^b^ Effect: The dietary treatment (D), time (S), and the interaction between dietary treatment × time (D × S) effects were analyzed by ANOVA using a general linear model (GLM) for repeated measures, and posthoc analysis was performed with appropriate use of Tukey’s multiple range test.

**Table 5:** Enzyme activities (Units/mL), total antioxidant capacity, and oxidative status biomarkers in blood plasma and milk of ewes fed diets (CON, CSS6, CSS11, CSS16) with different levels of Camelina seeds (6%, 11%, and 16% of concentrate) throughout the experimental period (15th, 30th, 45th, and 60th experimental days).

	Dietary Treatment (D)					Sampling Time (S)					Effect ^b^		
	CON	CSS6	CSS11	CSS16	SEM ^a^	15	30	45	60	SEM ^a^	D	S	D × S
Blood Plasma													
SOD	14.44 ^a^	15.57 ^ab^	15.98 ^ab^	16.91 ^b^	0.426	14.36 ^A^	14.91 ^B^	17.05 ^C^	16.57 ^C^	0.400	0.014	<0.001	0.109
CAT	19.37 ^a^	22.17 ^a^	22.93 ^b^	22.18 ^b^	0.522	21.46 ^A^	22.94 ^B^	21.17 ^A^	21.10 ^A^	0.556	0.006	0.052	0.536
GSH-Px	0.24	0.26	0.28	0.27	0.009	0.33 ^D^	0.27 ^C^	0.24 ^B^	0.22 ^A^	0.007	0.112	<0.001	0.189
GR	0.05	0.05	0.05	0.06	0.001	0.049 ^A^	0.054 ^B^	0.051 ^A^	0.054 ^B^	0.001	0.102	0.011	<0.001
GSTs	0.15 ^t^	0.17 ^a^	0.17 ^a^	0.19 ^t^	0.009	0.14 ^A^	0.19 ^B^	0.19 ^B^	0.16 ^A^	0.009	0.137	<0.001	0.025
ABTS	30.10 ^a^	32.81 ^b^	33.52 ^b^	31.01 ^a^	0.554	31.04 ^B^	34.70 ^C^	31.79 ^B^	29.91 ^A^	0.518	<0.001	<0.001	<0.001
FRAP	0.93 ^a^	0.91 ^a^	0.97 ^ab^	1.04 ^b^	0.028	1.00 ^B^	0.99 ^B^	0.88 ^A^	0.98 ^B^	0.028	0.008	0.001	<0.001
MDA	0.63 ^ac^	0.61 ^a^	0.74 ^b^	0.70 ^cb^	0.021	0.66	0.66	0.69	0.66	0.022	<0.001	0.516	<0.001
PC	2.45 ^a^	2.24 ^a^	2.47 ^a^	3.08 ^b^	0.213	3.35 ^C^	2.71 ^BC^	2.31 ^AB^	1.87 ^A^	0.195	<0.001	<0.001	<0.001
Milk													
SOD	131.49 ^a^	142.76 ^ab^	141.37 ^ab^	149.63 ^b^	1.685	131.78 ^A^	143.27 ^BC^	140.11 ^B^	150.09 ^C^	3.245	0.019	0.006	0.109
CAT	3.68 ^a^	5.16 ^b^	5.96 ^b^	7.85 ^c^	0.359	5.70 ^B^	7.02 ^C^	4.45 ^A^	5.49 ^B^	0.391	<0.001	<0.001	0.007
GSH-Px	0.28 ^a^	0.32 ^b^	0.31 ^b^	0.32 ^b^	0.007	0.31 ^B^	0.33 ^C^	0.27 ^A^	0.32 ^BC^	0.007	<0.001	<0.001	<0.001
ABTS	48.04 ^ab^	50.32 ^b^	53.36 ^c^	54.46 ^bc^	1.412	54.71 ^B^	45.99 ^A^	47.02 ^A^	58.46 ^C^	1.221	0.001	<0.001	<0.001
FRAP	3.00 ^a^	4.77 ^b^	5.21 ^bc^	5.85 ^c^	0.302	4.33 ^B^	3.56 ^A^	3.66 ^A^	7.27 ^C^	0.261	0.008	0.001	<0.001
MDA	0.24 ^ab^	0.25 ^ab^	0.26 ^b^	0.21 ^a^	0.244	0.21 ^A^	0.25 ^B^	0.27 ^BC^	0.22 ^AB^	0.242	0.036	0.023	0.074
PC	1.71 ^b^	1.63 ^b^	1.60 ^b^	1.28 ^a^	0.086	1.20 ^A^	1.77 ^B^	1.68 ^B^	1.57 ^B^	0.080	<0.001	<0.001	<0.001

Means with different superscript letters (a, b, c) between dietary groups and (A, B, C, D) between sampling time points differ significantly. ^a^ SEM: Standard error of the means. ^b^ Effect: The dietary treatment (D), time (S), and the interaction between dietary treatment × time (D × S) effects were analyzed by ANOVA using a general linear model (GLM) for repeated measures, and post-hoc analysis was performed with appropriate use of Tukey’s multiple range test. FRAP is expressed as μM ascorbic acid equivalents, ABTS as% inhibition, MDA as μM MDA, and PC as nmol/mL.

## Data Availability

All data are contained within the article and supplementary file.

## Supplementary Materials

The following are available online at https://www.mdpi.com/article/10.3390/foods10092076/s1. Supplementary file.
