# Supplementary material for: Assessing the Optimum Level of Supplementation with Camelina Seeds in Ewes’ Diets to Improve Milk Quality
Source: Foods. 2021 Sep 2;10(9):2076. doi: 10.3390/foods10092076 (PMC8465129; doi:10.3390/foods10092076)
Supplement: Supplementary file 1 [file foods-10-02076-s001.zip › foods-1348876-supplementary.pdf]

foods

Enzyme assays in both blood plasma and milk

Glutathione transferase activity in blood plasma was measured according to the method described by Labrou, Mello, and Clonis (2001). Briefly, 150 μl of blood plasma was added to 850 μl working solution (pH 6.5), consisted of 784 μl potassium phosphate (Alfa Aesar), 33 μl reduced glutathione with concentration 75 mm (Alfa Aesar) and 33 μl 1-chloro-2,4-dinitrobenzene with concentration of 30 mm (Alfa Aesar) and the absorbance was recorded at 340 nm for 2 min.

Catalase activity in blood plasma was assessed using continuous spectrophotometric rate for the determination of H2O2 at 520 nm, according to Sigma-Aldrich Catalase Assay Kit (CAT100).

Glutathione peroxidase activity in blood plasma was measured according to the method of Paglia and Valentine (1967). More specifically, 50 μl blood plasma was added to 650 μl working solution (pH 7.0), consisted of 499 μl potassium phosphate buffer with concentration 100 mm (Alfa Aesar), including 1 mm EDTA (Panreac ITW Companies), 8 μl of 1 mm glutathione reduced (Alfa Aesar), 30 μl of 0.042% hydrogen peroxide (Sigma-Aldrich, CO, USA) and 12 μl NADPH tetrasodium salt with concentration 0.2 μm (Alfa Aesar). Then, 1 μl of GRD (glutathione reductase, Sigma-Aldrich) was added to the cuvette and the absorbance was recorded at 340 for 5 min.

Glutathione reductase activity in both blood plasma and milk was measured according to the method of Mavis and Stellwagen (1968). Briefly, 100 μl of blood plasma and/or milk was added to 900 μl working solution (pH 7.6), consisted of 500 μl potassium phosphate with concentration 100 mm (Alfa Aesar), 33 μl oxidized glutathione (30 mm) (Sigma-Aldrich), 117 μl NADPH tetrasodium salt (0.8 mm) (Panreac ITW Companies), 33 μl of 1% bovine serum albumin (Alfa Aesar), 217 μl double distilled water, and the reduction in the absorbance was recorded at 340 for 3 min.

Superoxide dismutase activity in both blood plasma and milk was assayed using the method of McCord and Fridovich (1969) after modifications. More specifically, 50 μl blood plasma and/or milk was added to 917 μl of a reaction cocktail, comprised of 216 mm potassium phosphate (Alfa Aesar), 10.7 mm EDTA (Panreac ITW Companies), 1.1 mm cytochrome c (Sigma-Aldrich) and 0.108 mm xanthine (Alfa Aesar) and before the monitoring at 550 nm for 5 min, 33 μl of xanthine oxidase (Sigma-Aldrich) at 0.05 units/ml was added.

Lactoperoxidase activity in milk was measured according to the methods of Keesey (1987) and Putter and Becker (1983). Briefly, 20 μl of defatted milk was added to 980 μl working solution (pH 5.5), consisted of 714 μl 50 mm potassium phosphate with concentration of 50 mm (Alfa Aesar), 233 μl ABTS and 33 μl of 0.025% hydrogen peroxide (Sigma-Aldrich) and then the increasing of the absorbance was monitored at 340 for 3 min.

Antioxidant and free radical scavenging activities

The 2,2-azino-bis(3-ethylbenzothiazoline-6-sulphonic acid) (ABTS) radical scavenging assay was based on the published methods (Pellegrini et al., 2003; Li et al., 2011). Briefly, the stock solution of the ABTS radical was prepared by dissolving 3.84 mg of 2,2-azinobis(3-ethylbenzothiazoline-6-acid, Alfa Aesar) in 1 ml of water. The ABTS was dissolved in 1 ml of potassium persulphate 2.45 mm (Sigma-Aldrich), and the mixture was stored in the dark for 12 hr. The working solution was obtained by diluting the stock solution of ABTS radical cation with phosphate buffer saline (PBS) 0.005 m and pH 7.4 to obtain an absorbance of 0.7 ± 0.005 at 734 nm. Blood samples were diluted properly and 10 μl of dilution reacted with 990 μl ABTS working solution. Absorbance was measured at 734 nm against the reference

sample (PBS). The antioxidant capacity of blood samples was expressed as % inhibition. Ferric reducing ability of plasma (FRAP) assay was used to measure total antioxidant potential according to the method described by Benzie and Strain (1996). Three solutions of 300 mm CH3COONa, 10 mm 2,4,6-tripyridyl-S-triazine (Sigma-Aldrich) and 20 mm FeCl3 (Alfa Aesar) were prepared and mixed 10:1:1, respectively. 990 μl of FRAP solution and 10 μl of blood plasma were mixed and left at 37°C for 4 min. Absorbance was measured at 593 nm. Dilutions of the samples were made accordingly. Ascorbic acid was used as standard. The same protocols were used for milk samples for the determination of FRAP and ABTS, but with some modifications. More specifically, for the extraction procedure, one normal solution of HCl (1 N)/95% ethanol (v/v, 15/85) (Merck KGaA) was prepared and used as an extraction solvent. Then, 1 ml of the fresh milk was added to 10 ml solvent in 50-ml brown bottles and was shaken for 1 h at 30°C in a rotary shaker set at 300 rpm. The mixture of solvent and samples was then centrifuged at 7,800 g at 5°C for 15 min. The supernatant fluids were kept at −20°C in the dark until further analysis of FRAP and ABTS.

Lipid peroxidation activity and protein carbonyl determination in both blood plasma and milk

Lipid peroxidation activity was assayed by measuring MDA according to the method of Nielsen, Mikkelsen, Nielsen, Andersen, and Grand-Jean (1997) with some modifications. More specifically, 100 μl blood plasma was added to 700 μl of 1% ortho-phosphoric acid (Panreac ITW Companies) and 200 μl of 0.6% aquarius thiobarbituric acid (TBA, Sigma-Aldrich) and then the samples were heated at 100°C for 60 min. In milk samples, 1 mL of raw milk was added to 7 mL of 1% ortho-phosphoric acid (Panreac ITW Companies) and 2 mL of 0.6% aquarius TBA (Sigma-Aldrich) and then incubated at 100°C for 60 min. After that, the samples were filtered from cellulose acetate (MACHEREY-NAGEL) microfilter 0.45 μm and the absorbance recorded at 532 nm.

Protein carbonyls contents were determined based on a published method (Patsoukis et al., 2004) as described by Matthaiou et al. (2014) with the same quantities both for blood plasma and milk. More specifically, 50 μl of 20% aquarius TCA (Trichloroacetic acid, Sigma-Aldrich) was added to 50 μl of plasma and/or milk and this mixture was incubated in an ice bath for 15 min and centrifuged at 15,000 g for 5 min at 4°C. The supernatant was discarded, and 500 μl of 10 mm 2,4-dinitrophenylhydrazine (DNPH, Sigma-Aldrich) (in 2.5 N hydrochloride [HCl]) for the sample, or 500 μl of 2.5 N HCl for the blank, was added in the pellet. The samples were incubated in the dark at room temperature for 1 hr, with intermittent vortexing every 15 min, and were centrifuged at 15,000 g for 5 min at 4°C. The supernatant was discarded, and 1 mL of 10% aquarius TCA was added, vortexed and centrifuged at 15,000 g for 5 min at 4°C. The supernatant was discarded, and 1 mL of ethanol–ethyl acetate (1:1 v/v) (Merck KGaA) was added, vortexed and centrifuged at 15,000 g for 5 min at 4°C. This washing step was repeated twice. The supernatant was discarded, and 1 mL of 5 m urea (pH 2.3) was added, vortexed and incubated at 37°C for 15 min. The samples were centrifuged at 15,000 g for 3 min at 4°C, and the absorbance was read at 375 nm. Calculation of PC concentration was based on the molar extinction coefficient of DNPH.
